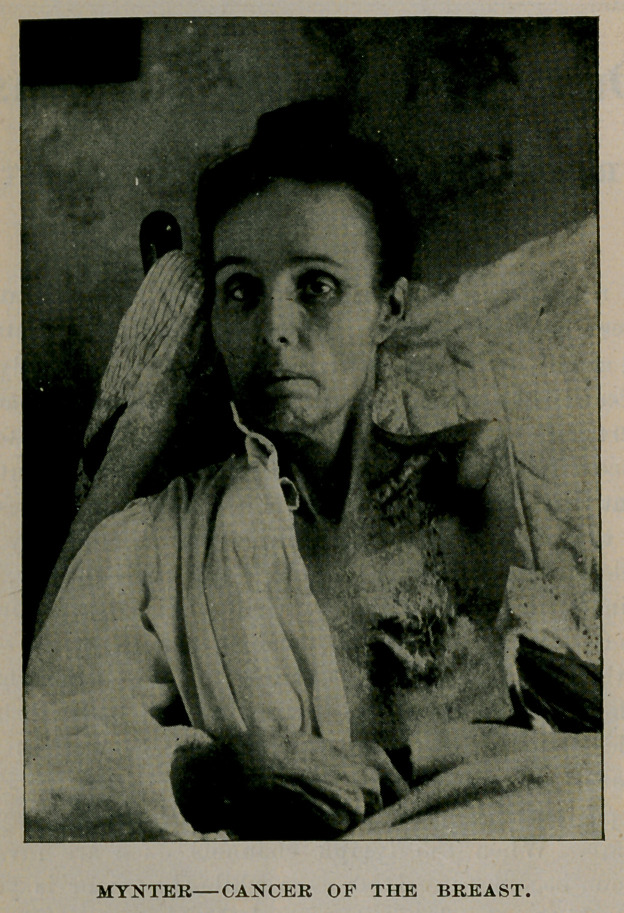# Remarks on Cancer of the Breast

**Published:** 1895-08

**Authors:** Herman Mynter

**Affiliations:** Buffalo, N. Y., Professor of surgery in Niagara University.


BUFFALO MEDICAL JOURNAL.

VOL. XXXV. AUGUST, 1895. No. 1.

### Original Communications.

### REMARKS ON CANCER OF THE BREAST.

BY HERMAN MYNTER, M. D., Buffalo, N. Y.,
Professor of surgery in Niagara University.

THE results of operations for cancer of the breast lately have been much improved. While Gross and Agnew stated that they had never cured a case and that they operated solely for the moral effect on the patient, the latest statistics of Halstead show a cure of 94 per cent. Even if his cases, perhaps, are of too recent occurrence to build reliable statistics upon, the improvement is surely so evident that it behooves us to inquire into the causes and we will find that, as in appendicitis, the decreased mortality depends upon a clearer knowledge in pathology and, consequently, a radical change in operative procedures. While, perhaps, the proof that cancer is an infectious disease dependent upon germs is still lacking and the germs or cells still unknown, it is well recognised that it is in the start a local disease, infecting the body through the lymphatics and that, therefore, an early operation offers the best chance for a radical cure.

The pectoralis muscle becomes affected early, according to Haidenhain. When the lymph channels once are invaded, the muscle soon becomes involved, even while the tumor is yet freely movable. If the muscle once is attacked, the cancer cells, or whatever they be, spread by muscular contractions through the whole muscle.

The former belief was that all the lymphatics passed through the axillary glands. Volkmann and others, however, have demonstrated that there is an intricate network of lymphatics on the surface of the pectoralis major muscle and upon the upper side of the fascia ; even that there is a connection of the lymphatics of the right and left breast; that the lymphatics under the pectoralis major and minor in the subclavicular region, in the sulcus bicipitalis, along the axillary vessels and other places may become infected. All these regions have to be considered in a modern operation for cancer of the breast, and it is no longer sufficient to remove the tumor and the axillary glands.

MYNTER—CANCER OF THE BREAST.

The not infrequent recurrence, with increased malignity, following an amputation of the breast is probably the result of rough handling of the tumor during the operation, by which cancer cells are forced into the lymphatics. The earlier operation consisted simply in extirpating the tumor.

Küster was the first to advocate cleaning out the axilla in every case. Volkmann went a step further in removing the fascia covering the pectoralis muscle. Gerster proposed to attack the axilla first in order to cut off the lymph channels before handling the tumor.

Two American surgeons, Halsted[1] and Willy Meyer,[2] have during the last year, independently of each other, advocated a more radical operation, removing in one piece pectoralis major and minor, the whole breast, the contents of the axilla and the sub- and infraclavicular region. Of these two operations I consider Meyer's the easiest and anatomically the most correct, as the lymph channels are cut through at the beginning of the operation, and infection from handling the tumor is prevented. The loss of the pectoralis major muscle does not seriously interfere with the use of the arm as the anterior portion of the deltoid muscle partly compensates the lost muscle. The whole breast should be removed in each case, no attention being paid to the loss of skin. In two recent cases I successfully skingrafted the enormous defect down on the very ribs, at the time of operation, and the patients left the hospital recovered in about two weeks.

As long as a recurrence can be removed by the knife it ought to be done, provided there is no systemic affection of internal organs. There is almost no limit to operative proceedings, life will be prolonged even if a cure is not obtained and much misery and suffering avoided. In this connection I wish to report a case:

Mrs. H., 53 years of age. She had her left breast removed by caustics nine years ago. One year later she came to me with a large relapse in the scar and glandular enlargement in the axilla. I made as thorough an operation as was possible at that time. She has since had six relapses in the axilla, each of which I removed with more and more difficulty on account of the cicatricial retraction of the axillary tissues, the last operation being done in October, 1893. She again entered the Sisters' Hospital in March, 1895, in a deplorable condition, the left arm being three times the size of the right on account of pressure of a relapse on the axillary vein. The axilla was occupied by a hard immovable tumor as large as a fist and inoperable by the usual methods. A hard infiltration was felt under the clavicle up along the nerves and vessels. She suffered excruciating pain from the affection of the nerves and was obliged to take morphine in large doses continually. She was willing to submit to any operation by which her life could he lengthened and her misery abated. It was impossible to get at the tumor on account of the enormously swollen arm, which could not be abducted on account of the scars in the axilla, and it was necessary, therefore, to exarticulate the arm as a preliminary operation.

Having first ligated the subclavian artery and vein in their third division I exarticulated the arm, saving only a large outside flap consisting of skin and deltoid muscle. I thereafter removed the tumor and all contents of the axilla, skin included, cleaned out completely the infraclavicular space with the infected nerves and vessels, removed the pectoralis muscles and covered the axillary defect with the deltoïd flap. The operation was absolutely bloodless. The patient left the hospital recovered in fourteen days, relieved of the pain, eating well and rapidly increasing in strength. [See illustration, p. 2.]

**I have never heard of this operation having been done before and, therefore, report it here.**

1. *Annals of Surgery*, November, 1894.
2. *Medical Record*, December 15, 1894.

566 DELAWARE AVENUE.

## Figures and Tables

**Figure f1:**